# The Physician’s Pocket Day Book

**Published:** 1884-01

**Authors:** 


**The Physician's Pocket Day Book.** Designed by C. HENRI LEONARD, M. A., M. D., Professor of Medical and Surgical Diseases of Women in the Michigan College of Medicine. Price $1.00. Illustrated Medical Journal Co., Detroit, Mich.

The diary accommodates daily charges for twenty or forty families weekly, has complete obstetrical record for ninety-four cases and monthly memorandum for debtor and creditor cash account. It is one of the most convenient to carry in the pocket, being of convenient shape and small bulk.

